# The perceived impacts of United States foreign development assistance reductions on health system building blocks at healthcare facilities in Zambia: A mixed-methods study

**DOI:** 10.1371/journal.pgph.0006090

**Published:** 2026-03-16

**Authors:** Lucy K. Tantum, Darcy M. Anderson, Hikabasa Halwiindi, Michael E. Herce, Michael T. Mbizvo, Miles A. Kirby, Ryan Cronk

**Affiliations:** 1 Department of Environmental Sciences and Engineering, Gillings School of Global Public Health, The Water Institute at UNC, The University of North Carolina at Chapel Hill, Chapel Hill, North Carolina, United States of America; 2 Department of Community and Family Medicine, School of Public Health, University of Zambia, Lusaka, Zambia; 3 Centre for Infectious Disease Research in Zambia (CIDRZ), Lusaka, Zambia; 4 Institute for Global Health and Infectious Diseases, University of North Carolina, Chapel Hill, North Carolina, United States of America; 5 Population Council, Lusaka, Zambia; 6 World Vision United States of America, Federal Way, Washington, United States of America; Worcester Polytechnic Institute, UNITED STATES OF AMERICA

## Abstract

The rapid reduction in foreign development assistance from the United States and other countries in 2025 has disrupted essential global health programming. Countries that previously received development assistance, such as Zambia, may experience weakening of health system capacity due to program cancellations. This study aimed to describe the perceived impacts of USAID program defunding on healthcare delivery in Zambia. We conducted a cross-sectional survey at 34 purposively selected healthcare facilities in three districts of Zambia in April and May 2025. Through facility-level assessments and individual-level surveys with 330 healthcare workers, we identified changes in health system building blocks that may have arisen from program defunding. The facility-level assessment found that 71% of healthcare facilities (n = 24) experienced changes related to funding cuts within the previous three to four months. In open-ended surveys, healthcare workers reported impacts on the health system, including stock-outs of essential medicines, diagnostic tests, and infection control supplies; layoffs of US-supported healthcare workers; and reduced ability to work with electronic medical records. Workers described how these changes affected workplace morale, patient satisfaction, and their ability to deliver essential services. This study reveals the immediate consequences of defunding foreign assistance, which, if left unaddressed, may weaken health systems and worsen health outcomes. National governments and partner organizations should prioritize interventions and investments that build health system resilience, such as expanding healthcare revenue streams and improving workforce capacity. In the wake of funding cuts, health system strengthening can reduce reliance on foreign assistance and improve population health.

## Introduction

United States official development assistance has been an important source of economic support for global health, comprising 29% of all health development assistance in low- and middle-income countries in 2023 [1]. US development assistance has supported a wide range of global health initiatives, including programs addressing maternal and child health, HIV/AIDS, malaria, tuberculosis, and health system strengthening [2]. In January 2025, the US federal government announced a pause in foreign development assistance and began suspending or eliminating United States Agency for International Development (USAID) programs [3]. Subsequently, in March 2025, the Trump administration announced the cancellation of 83% of USAID programs and the intent to dissolve the Agency [4,5]. A small proportion of these programs was later reinstated through the US State Department [6,7]. Following the US funding cuts, several European countries also reduced their foreign assistance programs, resulting in an overall estimated 22% drop in development assistance for health from 2024 to 2025 [8,9].

This reduction in foreign assistance funding has had widespread implications for health systems globally [10,11]. USAID-supported organizations have described immediate disruptions to health-related programming, including worker layoffs, stockouts of essential medicines and medical supplies, and reduced ability to deliver services such as immunization and diagnostic testing [8,12,13]. In the long term, if gaps in funding are not addressed, foreign assistance cuts will lead to gaps in service delivery that could increase global mortality and reverse progress toward universal health coverage [14–16]. A forecasting analysis estimated that reductions in USAID funding could lead to over 1.7 million deaths in all ages and over 600,0000 deaths in children under age 5 by 2030, drawing from estimates of USAID’s health impacts across all program areas [17]. Another analysis estimated that, due to foreign aid cuts, over 200,000 additional child deaths would occur globally in 2025 compared to 2024 [18]. Other models have projected that foreign assistance reductions could result in over 50,000 excess child deaths from malaria, over 126,000 excess child deaths from diarrhea, and over 159,000 excess adult deaths from HIV in one year [19].

Indirect impacts of reductions in foreign assistance may arise from changes at the healthcare facility level. Through its programming for global development and maternal-child health, USAID has supported infectious disease testing and treatment, health workforce development, and healthcare facility infrastructure improvement, including programs advancing water, sanitation, and hygiene (WASH) [20,21]. Reduced WASH infrastructure provision in healthcare facilities may increase the risk of healthcare-associated infections, prolonging illness and driving up healthcare costs [22,23]. Impacts on the quality and timeliness of healthcare may also arise from shortages of essential medicines and healthcare worker layoffs. Studies of healthcare utilization during epidemics indicate that substandard conditions at facilities may also lead patients to delay or avoid seeking healthcare [24,25].

The effects of defunding will disproportionately impact health systems in countries that were previously receiving large amounts of USAID funding, including Zambia [9]. In the 2023 fiscal year, USAID allocated $360 million to programs in Zambia, including $188 million for the HIV/AIDS sector, $66 million for the basic health sector (including tuberculosis, malaria, and nutrition), and $20 million for maternal-child health and family planning [26]. In comparison, at the time of this publication, USAID had allocated $135 million to programs in Zambia in the 2025 fiscal year, and recissions may contribute to further cuts [4,26]. As foreign development assistance comprises a large portion of its national health budget, overall health spending in Zambia is projected to decline by 10–15% in 2025 compared to 2022 [9]. Funding reductions may impede health system strengthening in Zambia. Despite efforts to recruit and train healthcare workers, Zambia still faces a severe healthcare worker shortage, with an estimated 1:12,000 doctor-to-patient ratio (compared to an ideal ratio of 1:5000), and 1:14,900 nurse-to-patient ratio (compared to an ideal ratio of 1:700) [27]. Many healthcare facilities lack essential infrastructure: a 2024 UNICEF report estimated that, among non-hospital facilities in Zambia, 68% had basic water services, 1% had basic sanitation services, and 13% had basic waste management services in 2023 [28].

According to the World Health Organization, strong health systems require a set of core “building blocks,” including regulatory mechanisms, financial and human resources, and support for healthcare delivery [29]. In health systems where programs have received support from foreign development assistance, funding cuts may impact one or more of these building blocks. To date, however, few studies have collected and reported primary data on the health system impacts of foreign assistance funding cuts in Zambia or other low- and middle-income settings. Research on this topic can inform strategies to maintain continuity in healthcare delivery despite variable funding. The objectives of this study were to describe how reductions in foreign development assistance have contributed to changes at healthcare facilities in Zambia, and assess the potential implications of these changes for the health and well-being of patients and healthcare workers.

## Methods

### Study design

This exploratory, descriptive mixed-methods study assessed health service delivery in Zambian healthcare facilities shortly after USAID program suspensions began. A research team surveyed staff at healthcare facilities and evaluated facility conditions to identify changes that occurred during this time period. Data collection took place from April 26 to May 28, 2025, three to four months after the US government announced the initial USAID funding freeze and program review [3]. This study was part of a broader evaluation of WASH and healthcare worker well-being at healthcare facilities in Zambia.

With data from a facility-level assessment, we characterized the reported impacts of defunding on facility operations and described the availability of WASH services, essential medicines, and medical equipment. We also analyzed data from open-ended surveys with individual healthcare workers to assess changes in health system inputs. Using the World Health Organization (WHO) Building Blocks of Health Systems framework, we classified changes reported in healthcare worker surveys that related to one or more building blocks: service delivery, health workforce, access to essential medicines, health information systems, financing, and governance [29].

### Study sites and population

All government-run healthcare facilities in Luwingu District and Lupososhi District, Northern Province, and Lusaka District, Lusaka Province, Zambia were included in the study sampling frame. These districts were purposively selected for an evaluation of programs that had been implemented by the non-governmental organization World Vision. We selected districts that had previously received the World Vision program or could serve as a non-program comparison group. Within each district, we purposively selected 9–13 healthcare facilities based on urban/rural status and facility type to obtain data from a range of healthcare contexts. Eligible facility types included health posts, health centers, mini hospitals (which offer diagnostic services, minor surgical services, and inpatient care), and first-level hospitals (which offer medical and surgical services and receive referrals from mini hospitals and health centers) [30]. All of the selected healthcare facilities agreed to participate.

At each facility, we purposively selected one knowledgeable staff member, such as a medical director or superintendent, nurse in-charge, or environmental health technician, to participate in a facility-level assessment of infrastructure and service provision. When necessary, we consulted additional staff for information that the original respondent was unable to provide. We selected additional healthcare workers to participate in individual-level surveys. All individuals employed by the healthcare facility, including clinical and non-clinical staff, were eligible to participate in the individual-level surveys. Many of the rural health posts and clinics in our sample had fewer than 10 staff members present on the day of data collection; at these facilities, we selected all available staff to participate in the survey. At larger healthcare facilities with more staff, we used purposive and convenience sampling to select individuals based on their staff role, clinical department, and availability to participate. The research team visited each department and invited employees in various roles to participate in the survey. At these larger facilities, the target sample size was 15–20 participants.

### Data collection

Data collection comprised a facility-level assessment followed by an individual-level survey of healthcare workers. First, for the facility-level assessment, an American or Zambian researcher assessed service delivery by speaking with a knowledgeable staff member and directly observing WASH infrastructure, including water systems, hand hygiene stations, toilets, and waste management facilities, at each selected healthcare facility. The assessment included questions on the types of healthcare services provided and typical patient volumes; the availability and functionality of WASH infrastructure; and the availability of essential medicines and equipment. We determined the availability of seven essential medicines (ciprofloxacin, amoxycillin, paracetamol, oral rehydration salts, any first-line tuberculosis treatment, any first-line antimalarial, and any antiretroviral) and six medical equipment (weighing scale, thermometer, stethoscope, blood pressure cuff, refrigerator, and computer with internet access), drawing from WHO guidelines for essential medicines and service delivery indicators [29].

The facility-level assessment also asked a question about US foreign development assistance reductions: “Has the facility experienced changes due to recent USAID funding cuts?” If respondents answered affirmatively, researchers asked an open-ended question about the type of impact experienced and coded the best-fitting response category on the survey form. The response categories were developed during a survey pre-test at three healthcare facilities. During pre-testing, we collected preliminary information about perceived impacts at facilities through open-ended survey questions and informal discussions with staff. Researchers could also select an “other” category and write a text description for responses that did not fit into any categories. Researchers verbally administered all assessment questions and recorded responses in a Kobo Toolbox form on a tablet.

In addition to the facility-level assessment, we collected data on changes in healthcare facilities through surveys administered to individual healthcare workers. Survey questions were part of a larger assessment of WASH-related well-being among healthcare workers. During individual-level surveys, Zambian researchers verbally administered an initial, open-ended question about whether the facility had experienced any changes since the start of the foreign development assistance freeze in January 2025. Depending on the participant’s response, researchers then asked additional open-ended probing questions. Researchers were given a list of probing questions and independently decided which, if any, to ask the participant. These optional probes asked whether the availability of essential medicines or WASH services had changed; whether any changes had resulted from USAID defunding; and how these changes had affected worker and patient well-being. However, the survey form did not direct researchers to include all components of the health system building blocks. Participants responded verbally, and researchers transcribed or paraphrased responses in text using a Kobo Toolbox form.

### Data analysis

Using data from facility-level assessments, we generated descriptive statistics for facility characteristics, including facility type, size, and availability of essential medicines and equipment. We aggregated data on WASH infrastructure and supply availability to determine the proportion of facilities with a basic level of each WASH service, in accordance with criteria from the WHO/UNICEF Joint Monitoring Program [28]. This framework classifies each WASH service as “basic,” “limited,” or “no service” based on factors such as infrastructure type, location, and functionality [28]. Facility-level assessments also asked a knowledgeable staff member to report on changes that the facility experienced from USAID defunding. We used these assessment responses to characterize the types of impacts reported and to determine whether these impacts affected service delivery. When characterizing the types of impacts reported in the facility-level assessment, we re-categorized responses recorded as “other” by placing them into existing categories or creating new categories if needed. We used R software (Version 4.4.2) for statistical analysis.

Using data from open-ended questions in individual-level healthcare worker surveys, we conducted thematic analysis to assess health system elements that may be affected by USAID defunding. To classify changes reported by healthcare workers, we created a codebook with codes and definitions adapted from the WHO Building Blocks of Health Systems framework [29]. We classified each participant-reported change based on the health system building block to which it most closely corresponded. We developed additional codes to categorize downstream impacts on patient and healthcare worker health and well-being and to classify responses as no change or as changes unrelated to the health system. One researcher coded all survey responses using NVivo software (Version 14). Within each code, we reviewed and synthesized responses to describe how defunding of foreign development assistance may affect health system building blocks and patient/worker well-being at Zambian healthcare facilities.

### Ethics

At all healthcare facilities, we obtained permission from the medical director or another authorized official before initiating data collection. Healthcare workers provided written informed consent for participation in surveys. The study protocol was reviewed by the University of North Carolina IRB and was determined to be exempt from further review (reference no. 24–0733). The University of Zambia Humanities and Social Sciences Research Ethics Committee (HSSREC) IRB reviewed and approved the protocol (reference no. 00006464). The protocol also received clearance from Zambia’s National Health Research Authority, Ministry of Health, and District Health Offices for Luwingu, Lupososhi, and Lusaka.

Additional information regarding the ethical, cultural, and scientific considerations specific to inclusivity in global research is included in the Supporting Information (S1 Checklist).

## Results

### Facility-level characteristics and reported funding cut impacts

We assessed infrastructure and service delivery at 34 healthcare facilities across three districts of Zambia (Table 1). Facilities included health posts, health centers, mini-hospitals, and first-level hospitals. Sixty-two percent of facilities (n = 21) were in rural areas, while 38% (n = 13) were urban areas. Most facilities (59%, n = 20) provided both inpatient and outpatient services, with others providing only outpatient services. Most facilities had high volumes of outpatient visits, typically screening a median of 590 outpatients per month. Those that provided inpatient services typically admitted a median of 22 inpatients per month. In our evaluation of essential medicines and medical equipment availability, 29% percent of facilities (n = 10) had all medicines available, and 53% (n = 18) had all equipment available on the day of the assessment. On average, facilities had six of seven medicines available and five of six equipment available. Most facilities (62%, n = 21) used electricity from the national grid as their main energy source, while others primarily or solely used solar electricity. Facilities reported that they usually had electricity for between five and 24 hours each day, with a mean of 18 hours.

**Table 1:** Administrative characteristics and essential equipment and medicine availability at healthcare facilities in Zambia, 2025 (n = 34).

Healthcare facility characteristics	Number (percent)
*Basic characteristics*	
Facility type:	
Health center	14 (41.2%)
Health post	12 (35.3%)
First-level hospital	5 (14.7%)
Mini hospital	3 (8.8%)
Administrative district:	
Luwingu District	13 (38.2%)
Lusaka District	12 (35.3%)
Lupososhi District	9 (26.5%)
Location type:	
Rural	21 (61.8%)
Urban	13 (38.2%)
Services provided:	
Both inpatient and outpatient services	20 (58.8%)
Outpatient services only	14 (41.2%)
Typical number of inpatients admitted per month, median [Min, Max]	22 [5, 250]
Typical number of outpatients seen per month, median [Min, Max]	590 [60, 18000]
*Essential equipment, medicines, and infrastructure*	
6 out of 6 essential equipment* are available and functional	18 (52.9%)
7 out of 7 essential medicines** are available	10 (29.4%)
Primary energy source	
Grid electricity	21 (61.8%)
Solar electricity	13 (38.2%)

* Essential equipment assessed: Weighing scale, thermometer, stethoscope, blood pressure cuff, refrigerator, and computer with internet access.

** Essential medicines assessed: Ciprofloxacin, amoxycillin, paracetamol, oral rehydration salts, any first-line tuberculosis treatment, any first-line antimalarial, and any antiretroviral.

We assessed WASH service levels according to criteria from the WHO/UNICEF Joint Monitoring Programme for Water Supply, Sanitation, and Hygiene [28] (Table 2). Ninety-four percent of facilities (n = 32) had a basic water service, defined as an improved water source such as a piped water system, standpipe, or borehole, on facility premises. However, many facilities lacked continuous water service, with 41% (n = 14) reporting that water from their main source was available for fewer than 24 hours per day. Fifteen percent of facilities (n = 5) had basic sanitation services; these facilities had toilets or ventilated improved pit latrines with at least one dedicated staff toilet, at least one sex-separated toilet, and toilets accessible to individuals with limited mobility. A basic hand hygiene service, defined as functional hand hygiene facilities at all points of care and toilets, was available at 15% of facilities (n = 5). A basic waste management service, including safe segregation and disposal of sharps and infectious waste, was present at 26% of facilities (n = 9). Forty-one percent of facilities (n = 14) had a basic environmental cleaning service, defined as having cleaning protocols and training of all cleaning staff. One facility (3%) had a basic level of all five WASH services.

**Table 2:** WASH service levels at healthcare facilities in Zambia, 2025 (n = 34). WASH service levels are defined according to WHO/UNICEF Joint Monitoring Program criteria [28].

Healthcare facility WASH service levels	WASH service level definitions from WHO/UNICEF Joint Monitoring Program [28]	Number (percent)
*Water service*		
Basic	Water is available from an improved source (a source with the potential to deliver safe water) on premises.	32 (94.1%)
No service	Water is taken from unprotected dug wells or springs, or surface water sources; or an improved source that is more than 500 meters from the facility; or the facility has no water source.	2 (5.9%)
*Continuous water*	Water is available from the main source for 24 hours each day	19 (55.9%)
*Sanitation service*		
Basic	Improved sanitation facilities (facilities which can safely separate excreta from human contact) are usable. There is at least one toilet dedicated for staff, at least one sex-separated toilet with menstrual hygiene facilities, and at least one toilet accessible for people with limited mobility.	5 (14.7%)
Limited	At least one improved sanitation facility, but not all requirements for basic service are met.	29 (85.3%)
*Hand hygiene service*		
Basic	Hand hygiene facilities with water and soap and/or alcohol-based hand rub available at points of care and toilets.	5 (14.7%)
Limited	Functional hand hygiene facilities at either points of care or toilets, but not both.	26 (76.5%)
No service	No functional hand hygiene facilities at points of care or toilets.	3 (8.8%)
*Waste management service*		
Basic	Waste is segregated into at least three bins, and sharps and infectious waste are treated and disposed of safely.	9 (26.5%)
Limited	There is limited separation and/or treatment and disposal of sharps and infectious waste.	22 (64.7%)
No service	There are no separate bins for sharps or infectious waste, and waste is not treated/disposed of safely.	3 (8.8%)
*Environmental cleaning service*		
Basic	Protocols for cleaning are available and all cleaning staff have received training.	14 (41.2%)
Limited	There are cleaning protocols and/or at least some cleaning staff have received training.	17 (50.0%)
No service	No cleaning protocols are available and no cleaning staff have received training.	3 (8.8%)

In the facility-level assessment, 71% of respondents (n = 24) reported that their facility had experienced changes related to recent USAID funding cuts (Table 3). Among facilities that had experienced changes, the most common reported impact was on staffing levels: 42% (n = 10) of respondents reported that the number of non-medical staff at the facility had changed, and 33% (n = 8) reported that the number of medical staff had changed. Medical staff were defined as personnel who provided direct patient care (e.g., nurses, physicians, counselors), whereas non-medical staff were defined as personnel employed by the facility but who did not provide direct patient care (e.g., cleaners, laboratory staff, environmental health technicians). Other respondents described changes in the availability of essential medicines, supplies, and WASH services; patient health-seeking behavior and facility attendance; facility budgets; and the transport of laboratory samples. Eighty-three percent of respondents who reported changes (n = 20) said that these changes had affected the facility’s ability to deliver healthcare services.

**Table 3:** Changes reported at healthcare facilities in Zambia (n = 34) related to USAID defunding, 2025.

Changes experienced	Number (percent)
*Has the facility experienced changes due to recent USAID funding cuts?*	
Yes	24 (70.6%)
No	8 (23.5%)
Don’t know	2 (5.9%)
*Among the sub-set of respondents who reported changes: What type(s) of changes has the facility experienced?*	
Decrease in number of non-medical staff employed at the facility (e.g., cleaner)	10 (41.7%)
Decrease in number of medical staff employed at the facility (e.g., nurse)	8 (33.3%)
Decrease in availability of essential medicines	6 (25.0%)
Decrease in availability of WASH supplies and services	4 (16.7%)
Decrease in availability of medical supplies and equipment	2 (8.3%)
Decrease in patient attendance at the facility	2 (8.3%)
Decrease in facility’s overall operating budget	1 (4.2%)
Issues with transportation and logistics	1 (4.2%)
*Have these changes affected the facility’s ability to provide healthcare services?*	
Yes	20 (83.3%)
No	3 (12.5%)
Don’t know	1 (4.2%)

### Health system impacts reported by healthcare workers

**Fig 1:**
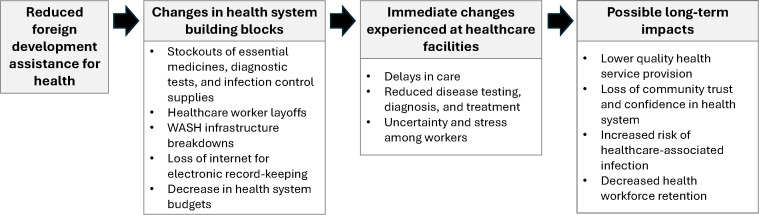
Conceptual framework for changes in health system building blocks, downstream changes occurring at healthcare facilities, and potential consequences for health arising from reductions in US foreign development assistance.

Healthcare workers (n = 330) participated in individual-level surveys about changes that had occurred at healthcare facilities from January 2025 through the day of data collection in April or May 2025. Survey participants represented a range of medical and non-medical staff roles (Table 4). Approximately half of the participants (n = 158, 48%) reported that no changes had occurred or only described changes unrelated to health systems. Among the 172 participants (52%) who described changes in one or more health system building blocks, many described detrimental changes that may have occurred in connection with USAID funding cuts. Some also described implications of these system-level changes for patient and worker well-being. Fig 1 depicts a conceptual model for changes in health system inputs, immediate changes at healthcare facilities reported by healthcare workers, and potential long-term impacts.

**Table 4:** Characteristics of participants (n = 330) in healthcare facility staff survey, Zambia, 2025.

Participant characteristics	Number (percent)
Role:	
Doctor, medical licentiate, or clinical officer	32 (9.7%)
Nurse	100 (30.3%)
Other medical staff	69 (20.9%)
Counselor or community health assistant	36 (10.9%)
Cleaner, maid, or general worker	56 (17.0%)
Other non-medical staff	37 (11.2%)
Gender:	
Female	218 (66.1%)
Male	112 (33.9%)
Healthcare facility type:	
Health center	148 (44.8%)
First-level hospital	113 (34.4%)
Health post	37 (11.2%)
Mini hospital	32 (9.7%)

#### Service delivery.

Interruptions in health service delivery occurred when facilities lacked sufficient materials or staffing to provide timely, comprehensive care. Facilities experienced shortages of test kits for HIV, malaria, and tuberculosis, and some participants reported that they had been unable to test patients for these conditions. Laboratory staff also reported shortages of consumable supplies for testing, such as specimen bottles. Timeliness of care was also impacted, with participants reporting that staff layoffs led to longer wait times for patients.

Healthcare facility infrastructure also affected service delivery. Many participants reported problems with WASH services at their facilities, including damage to toilets and ablution blocks, breakdowns of water pumps, and water pipe issues. These issues were most often reported at rural health posts and health centers. At some facilities, breakdowns in water or sanitation infrastructure remained unaddressed for several weeks or months. In other instances, participants reported that district-level health authorities had intervened to repair or install new systems. Rather than arising directly from changes in foreign development assistance, infrastructure breakdowns and repair delays may have been attributable to poor operations and maintenance practices that have been observed in low-income healthcare facility settings more generally [31].

“The water system no longer functions… It has affected patient care because sometimes you tell patients to go and wash hands outside instead of doing so within the building.” – Nurse, rural health center

#### Health workforce.

Healthcare facilities were affected by worker layoffs due to temporary or ongoing pauses in foreign development assistance. These layoffs particularly impacted staff in roles related to HIV care services in urban facilities, such as antiretroviral therapy (ART) clinic staff, peer educators, and contact tracers. Participants reported that these departments were now understaffed. In some instances, staff had been laid off and then reinstated, creating uncertainty about staffing levels and the ability to provide services consistently. Community-based health volunteers had received incentive payments from USAID for outreach work related to HIV/AIDS case-finding and treatment. Several participants at rural facilities reported that, after these payments were discontinued, volunteers no longer performed their duties, reducing community health engagement.

“When the USAID staff were stopped in February, the work got very messy...we depended on their system for so many things.” – Nurse, urban hospital

#### Access to essential medicines and supplies*.*

Many participants reported that issues with the availability of essential medicines had arisen since the start of 2025. Participants from multiple urban and rural healthcare facilities reported shortages of antimalarial, antiretroviral, and antibiotic medications, as well as family planning materials. In some cases, participants reported difficulties in distributing medicines according to the usual schedule or had to allocate smaller quantities to patients due to current or anticipated shortages. One participant reported that their facility lacked funding for transportation to collect medicines from distribution facilities. A May 2025 measure to reduce US funding for medicine procurement in Zambia, separate from USAID program cuts, may have also contributed to changes in medicine availability [32].

“The ARVs [antiretroviral drugs] given to the people above 40 and teenagers have been out of stock. They reduced from giving them for 6 months to 3 months due to anticipated shortage.” – Nurse, urban hospital

Participants also frequently reported shortages or stock-outs of WASH and infection control supplies, such as surgical gloves, hand soap, and hand sanitizer, at their facilities. Several cleaning staff also reported shortages of disinfectants and cleaning soaps, which affected their ability to perform their duties. It was unclear whether infection control supplies had previously been supplied through foreign development assistance programs.

“I am not doing my work like it should be done… Having a shortage of Jik [bleach] makes it difficult for my work.” – Cleaner, urban hospital

#### Health information systems.

USAID had supported internet services at some healthcare facilities, and several participants reported that their internet had recently been disconnected. Facilities relied on these internet services to enter electronic medical record data, and participants reported that working with records had become more cumbersome due to limited internet access. One participant said that their facility struggled to send health information system reports in a timely manner due to poor internet.

“The Starlink Internet from USAID recently stopped working, it’s been hard to work with records for the facility.” – Records clerk, rural mini hospital

### Financing and governance

Changes in health financing and governance structures were closely linked. Program cuts made by USAID, and, in turn, by other multilateral agencies and non-governmental organizations (NGOs) funded by USAID, led to reductions in funding at the healthcare facility level. In addition to layoffs of USAID-supported staff, a few participants reported decreases in external partnerships or in the receipt of monetary donations for their programs. Changes in external partnerships were more frequently reported at urban health centers and hospitals compared to rural facilities, potentially due to higher pre-2025 levels of donor participation at large, high-volume facilities.

“From the time [USAID] withdrew, all these NGOs stopped and affected the work.” – Nurse, urban health center

#### Implications for health and well-being.

Patients and healthcare workers experienced downstream changes from health system disruptions. Several participants expressed concern that patients were not receiving proper treatment due to shortages of essential medicines or testing supplies, potentially endangering health or prolonging illnesses.

“It makes us sad to see patients go back without proper treatment.” – Laboratory technician, urban health center

Layoffs of USAID-supported staff increased workloads for the remaining staff, and some participants reported feeling fatigued and stressed from attending to high patient volumes. Funding interruptions and layoffs reduced staff morale, with some expressing concerns about their future job security. Participants also reported increased fear of contracting infections due to the lack of infectious disease testing supplies, WASH supplies, and personal protective equipment at facilities.

“The status of water at the facility is not known. We are not sure if it is safe or contaminated because there’s no equipment for water testing.” – Laboratory technician, urban hospital

## Discussion

This study assessed changes at healthcare facilities in Zambia during the three to four months following the 2025 cuts to U.S. foreign development assistance programs, with healthcare workers reporting adverse impacts across all health system building blocks. Participants described difficulties in providing comprehensive, timely patient care and worries about their own health protection and job security due to the abrupt nature of the cuts. Staff roles and service delivery related to HIV services were particularly impacted by layoffs. Our assessment revealed that most facilities lacked one or more basic WASH services and experienced stockouts of essential medicines, underscoring the need for improved health infrastructure and strengthened supply chains. While Zambia’s health system contended with health workforce shortages and gaps in essential infrastructure prior to 2025 [27,28], foreign development assistance funding reductions may exacerbate these issues. Findings provide insight into the consequences of rapid, unplanned cuts in foreign development assistance for health system strengthening and global health security.

Our assessment of facility-level conditions and surveys of healthcare workers in Zambia identified shortages of diagnostic testing equipment, medicines, and staffing, which, if unaddressed, will negatively affect healthcare facilities’ ability to effectively treat patients, ultimately leading to prolonged illness and increased mortality. Previous assessments in Zambia have similarly identified a lack of preparedness for disease epidemics at healthcare facilities and in communities, suggesting that some of the reported shortcomings could pre-date foreign development assistance funding reductions [33,34]. These health risks will not be confined to low- and middle-income countries, as weakened health systems threaten global health security [35]. Epidemics and pandemics, such as Ebola and COVID-19, amplify and spread in healthcare facilities with little infection control infrastructure [36–38]. In addition to vulnerabilities identified at the country level, the US withdrawal from the World Health Organization will weaken WHO-coordinated surveillance and response networks, impeding the ability to track and contain epidemics [39]. Without coordinated response mechanisms, downstream consequences, within index countries and beyond, are likely to be severe and costly. National health security actors should consider supporting healthcare facility-level infection control infrastructure and global-level epidemic preparedness and response strategies for preventing the next epidemic or pandemic.

The gaps in WASH services that we identified in this study may pose a barrier to achieving development targets such as universal health coverage [40]. Under the 2022 US Global Water Strategy, USAID set objectives for expanding access to WASH services in households and healthcare facilities, including by building the capacity of local institutions to deliver and sustain services [41]. Improving and sustaining access to WASH in healthcare facilities pertains to the United Nations’ 2030 Sustainable Development Goals, including goals for good health and well-being (Goal 3) and universal coverage of clean water and sanitation (Goal 6) [42]. However, the 2025 foreign development assistance program cuts led to the suspension of WASH improvement projects in some areas, potentially hindering progress toward these objectives [43]. In addition to changes arising from program cuts, healthcare facilities in low-resource settings may struggle to plan and perform long-term maintenance of medical infrastructure and WASH services, undermining care delivery [31]. The development of plans and budgets for the installation, operations and maintenance of WASH infrastructure – including planning and budgeting at the facility, district, provincial, and national levels – could help to sustain service delivery.

Strategies to increase health system revenue and resilience could mitigate some of the budgeting, health workforce, and supply chain issues identified in our study. The government of Zambia has sought to achieve universal health coverage through a strategic plan that includes health workforce expansion, supply chain strengthening, health infrastructure upgrades, and decentralization of services [44,45]. However, 42% of Zambia’s per capita health expenditure was donor-funded in 2021; abrupt reductions in external financing will hamper the government’s ability to make and sustain health system investments [46]. Zambia’s National Health Insurance Management Authority has aimed to improve the quality of care through expanded service provision and essential medicine availability, but weak regulation and low public confidence pose barriers to uptake and quality [47–49]. Strengthened regulatory oversight of insurance schemes and public outreach could improve revenue generation from insurance, reducing dependence on external donor financing [50]. Furthermore, donor financing that targets specific disease areas under a vertical approach contributes to fragmented financing arrangements [50,51]. Integrated healthcare models, where service delivery is coordinated across health sector programs, can reduce costs of care while improving health outcomes [52,53]. Donor organizations, including USAID, have previously transitioned from vertical models to sector-wide approaches in some program areas [54,55], However, partnerships and coordination mechanisms may need to be revisited in light of funding reductions. By identifying areas of overlap across vertical programs and revenue streams, and moving toward greater integration, governments can improve the efficiency of health system operations and overall healthcare spending [54].

In addition to revenue generation, governments could explore resource allocation strategies to mitigate shocks from donor funding withdrawals. The government of Zambia could ring-fence domestic funding for a set of essential health system inputs, thereby ensuring that these funds are protected in the event of further external program disruptions [56]. This could include support for salaries of essential healthcare staff who were previously donor-funded (e.g., laboratory technicians, data staff, and community health workers) and for maintaining stocks of essential medicines and commodities. Resource allocation strategies should also account for the needs of primary care settings, which provide essential last-mile health services but may face weak supply chains and inadequate infrastructure [28,57]. To support care delivery in these settings, the government could implement facility-resilience packages that provide essential WASH services, energy infrastructure, and medical supplies.

Further research could also investigate the role of community outreach and engagement in maintaining continuity of care and reducing public health impacts of program cancellations. Some of the barriers to care delivery that were reported by our study participants – such as long waiting times, low availability of medicines, and poor quality of WASH services – have previously been described in studies of patient experiences in other healthcare settings [58,59]. Patients who encounter subpar services may feel hesitant to seek care in the future, undermining efforts to increase immunization, attended deliveries, and other facility-based services [60,61]. Efforts to build community trust in the health system and maintain service continuity could encourage health-seeking behavior, potentially moderating the health impacts of funding cuts [62,63].

This research is subject to limitations. In face-to-face surveys, participants may have either overstated or minimized the perceived impacts of foreign development assistance defunding due to social desirability bias. To minimize potential bias, surveys were administered in private locations within healthcare facilities, and researchers received training in impartial interviewing techniques. While interviewers were given a list of probing questions, they were not required to ask all of these questions, potentially introducing bias due to variations in interviewing techniques. We used a purposive sampling approach to select individuals for surveys and only conducted surveys during daytime hours; this approach may be subject to selection bias as we did not include workers who were unavailable, off-duty at the time of data collection, or had been laid off due to program cuts. This analysis was intended to assess healthcare worker perspectives on the impacts of foreign development assistance defunding. We did not verify whether the reported changes were directly attributable to USAID program cuts, nor did we collect longitudinal data to compare conditions before and after the cuts. Participants may have reported changes unrelated to US-funded programs, or changes they had heard about but not directly experienced. Because data collection occurred three to four months after the initial cancellations of U.S. foreign development assistance programs, we were unable to assess long-term changes. To better understand the link between defunding and health system impacts, future research could consult stakeholders involved with USAID programming across multiple levels of the health system in Zambia. Future projects could also employ quasi-experimental approaches to quantify the impacts of funding cuts on health service delivery indicators and health outcomes in affected areas.

## Conclusion

Healthcare facilities in Zambia have experienced interruptions to health service delivery following rapid reductions in US foreign development assistance. Recent studies have modeled the potential health impacts of foreign development assistance defunding or described changes experienced within specific programs [13,17,64], but to our knowledge, ours is the first to document changes occurring across multiple aspects of a health system at the facility level. Healthcare workers reported changes that affected their ability to deliver efficient and high-quality care, posing risks to patients’ health and eroding confidence in the healthcare system.

Despite funding reductions, our research suggests opportunities to rethink foreign development assistance priorities and to build domestic capacity in Zambia and other recipient countries. This study highlights the importance of sustained investment in WHO health system building blocks, especially supply chain strengthening and WASH infrastructure to support healthcare delivery. Health workforce capacity-building and integration of donor programs into the existing health system can strengthen health systems and support long-term program sustainability [65]. Country programs should also consider alternatives to conventional development assistance financing models, such as South-South collaboration, pan-African regional collaboration, and multilateral financing frameworks, to work toward shared development priorities. Initiatives to improve health system governance, coordination, and financing can insulate the health system against future disruptions and improve long-term health outcomes.

## Supporting information

S1 Checklist Inclusivity in global research questionnaire. (DOCX)

## References

[1] Dieleman JL, Apeagyei AE, Hay SI, Mokdad AH, Murray CJL. The USA’s role in global development assistance for health, 2000–30. Lancet. 2024;404(10469):2258–60. 39645375 10.1016/S0140-6736(24)02266-9

[2] Tarnoff C. U.S. Agency for International Development (USAID): Background, Operations, and Issues. Congressional Research Service. 2015.

[3] The White House. The White House. 2025 [cited 2025 Jul 29]. Reevaluating And Realigning United States Foreign Aid. Available from: https://www.whitehouse.gov/presidential-actions/2025/01/reevaluating-and-realigning-united-states-foreign-aid/

[4] Kates J, Rouw A, Oum S. U.S. Foreign Aid Freeze & Dissolution of USAID: Timeline of Events [Internet]. KFF. 2025 [cited 2025 Jul 29]. Available from: https://www.kff.org/u-s-foreign-aid-freeze-dissolution-of-usaid-timeline-of-events/

[5] Linton C. CBS News. 2025 [cited 2025 Jul 29]. Secretary of state says 83% of USAID programs are being canceled. Available from: https://www.cbsnews.com/news/secretary-of-state-usaid-programs-canceled/

[6] Walker AS, Khurana M, Zhang C. What Remains of U.S.A.I.D.? The New York Times [Internet]. 2025 [cited 2025 Jul 29]; Available from: https://www.nytimes.com/interactive/2025/06/22/us/politics/usaid-foreign-aid-trump.html

[7] Rubio M. Next Steps on Building an America First State Department: Press Statement [Internet]. United States Department of State. 2025. Available from: https://www.state.gov/releases/office-of-the-spokesperson/2025/05/next-steps-on-building-an-america-first-state-department/

[8] International Council of Voluntary Agencies. Lives on the line: The Human Impact of US Foreign Aid Shifts [Internet]. Geneva: ICVA; 2025. Available from: https://www.icvanetwork.org/uploads/2025/03/Lives-on-the-Line-Final-Report.pdf

[9] Apeagyei AE, Bisignano C, Elliott H, Hay SI, Lidral-Porter B, Nam S, et al. Tracking development assistance for health, 1990-2030: historical trends, recent cuts, and outlook. Lancet. 2025;406(10501):337–48. doi: 10.1016/S0140-6736(25)01240-1 40680759 PMC12439094

[10] Dyer O. Relief agencies in shock as Trump cuts 90% of USAID funding. BMJ. 2025;388:r445. 10.1136/bmj.r445 40037664

[11] Mbah RE, Hardgrave CM, Mbah DE, Nutt A, Russell JG. The Impact of USAID Budget Cuts on Global Development Initiatives: A Review of Challenges, Responses, and Implications. ASSRJ. 2025;12(04):219–32.

[12] Physicians for Human Rights. “The System is Folding in on Itself”: The Impact of U.S. Global Health Funding Cuts in Kenya [Internet]. 2025 [cited 2025 Jul 25]. Available from: https://phr.org/wp-content/uploads/2025/07/PHR-Research-Brief-Aid-Cuts-Kenya-2025.pdf

[13] Ndjeka N, Kubjane M, Abdullah F, Mohr-Holland E, Subrayen P, Loveday M, et al. Impact of US funding cuts and stop work orders on TB services and research in South Africa. IJTLD Open. 2025;2(4):241–3. doi: 10.5588/ijtldopen.25.0168 40226132 PMC11984525

[14] Martinez-Alvarez M, Amouzou A, Barros AJD, Faye C, Behanzin P, Borghi J. Broken promises: the USA foreign aid freeze threatens women’s, children’s, and adolescents’ health. Lancet. 2025;405(10488):1448–50. 40222383 10.1016/S0140-6736(25)00558-6

[15] Menzies NA, Brown TS, Imai-Eaton JW, Dodd PJ, Cohen T, Martinez L. Potential pediatric tuberculosis incidence and deaths resulting from interruption in programmes supported by international health aid, 2025-2034: a mathematical modelling study [Internet]. medRxiv; 2025 [cited 2025 Sep 10]. 2025.05.29.25328574 p. Available from: https://www.medrxiv.org/content/10.1101/2025.05.29.25328574v1 10.1016/S2352-4642(25)00218-4 40953585

[16] Osendarp S, Ruel M, Udomkesmalee E, Tessema M, Haddad L. The full lethal impact of massive cuts to international food aid. Nature. 2025;640(8057):35–7. doi: 10.1038/d41586-025-00898-3 40140717

[17] Cavalcanti DM, De Oliveira Ferreira De Sales L, Da Silva AF, Basterra EL, Pena D, Monti C, et al. Evaluating the impact of two decades of USAID interventions and projecting the effects of defunding on mortality up to 2030: a retrospective impact evaluation and forecasting analysis. Lancet. 2025. 10.1016/S0140-6736(25)01186-9 PMC12274115 40609560

[18] Gates Foundation Goalkeepers. 2025 Goalkeepers Report [Internet]. Gates Foundation; 2025 [cited 2026 Jan 17]. Available from: https://www.gatesfoundation.org/-/media/goalkeepers/reports/2025-goalkeepers-report_en.pdf

[19] Nichols B, Moakley E. Impact Counter. [cited 2025 Sep 9]. Impact Dashboard - Impact Counter. Available from: https://impactcounter.com

[20] Sonderman KA, Citron I, Albutt K, Salaam-Blyther T, Romanzi L, Meara JG. USAID: Current support for global surgery and implications of reform. Surgery. 2018;164(6):1147–55. doi: 10.1016/j.surg.2018.05.074 30249431

[21] KFF. The U.S. Government and Global Maternal and Child Health Efforts [Internet]. KFF. 2022 [cited 2025 Aug 20]. Available from: https://www.kff.org/global-health-policy/the-u-s-government-and-global-maternal-and-child-health-efforts/

[22] Watson J, D’Mello-Guyett L, Flynn E, Falconer J, Esteves-Mills J, Prual A, et al. Interventions to improve water supply and quality, sanitation and handwashing facilities in healthcare facilities, and their effect on healthcare-associated infections in low-income and middle-income countries: a systematic review and supplementary scoping review. BMJ Glob Health. 2019;4(4):e001632. doi: 10.1136/bmjgh-2019-001632 31354976 PMC6626521

[23] Hutton G, Chase C, Kennedy-Walker R, Hamilton H. Financial and economic costs of healthcare-associated infections in Africa. J Hosp Infect. 2024;150:1–8. doi: 10.1016/j.jhin.2024.04.015 38723903

[24] Wagenaar BH, Augusto O, Beste J, Toomay SJ, Wickett E, Dunbar N. The 2014–2015 Ebola virus disease outbreak and primary healthcare delivery in Liberia: Time-series analyses for 2010–2016. PLOS Med. 2018;15(2):e1002508. doi: 10.1371/journal.pmed.1002508 PMC5819774 29462138

[25] Hategeka C, Carter SE, Chenge FM, Katanga EN, Lurton G, Mayaka SM-N, et al. Impact of the COVID-19 pandemic and response on the utilisation of health services in public facilities during the first wave in Kinshasa, the Democratic Republic of the Congo. BMJ Glob Health. 2021;6(7):e005955. doi: 10.1136/bmjgh-2021-005955 34315776 PMC8318723

[26] US State Department. ForeignAssistance.gov [Internet]. [cited 2025 Sep 10]. Available from: https://foreignassistance.gov/

[27] UNICEF Zambia. Zambia Health Budget Brief [Internet]. Lusaka, Zambia; 2024 [cited 2025 Sep 30]. Available from: https://www.unicef.org/zambia/media/4921/file/UNICEF%20ZAMBIA%20Budget%20brief_Health.pdf.pdf

[28] Joint Monitoring Programme. WASH in health care facilities 2023 data update: Special focus on primary health care. WHO/UNICEF Joint Monitoring Programme; 2024.

[29] World Health Organization. Monitoring the building blocks of health systems: a handbook of indicators and their measurement strategies [Internet]. Geneva: World Health Organization; 2010 [cited 2023 Apr 18]. Available from: https://apps.who.int/iris/handle/10665/258734

[30] WHO Zambia. Country cooperation strategy 2024-2027 [Internet]. World Health Organization Zambia; 2024 [cited 2025 Sep 30]. Available from: https://www.afro.who.int/sites/default/files/2025-04/WHO%20Zambia%20CCS%2024-27_%20Final%20pdf.pdf

[31] Tantum LK, Mahamane E, Bauza V, Mahamadou KOB, Sanoussi EY, Salzberg A, et al. Environmental Infrastructure Maintenance Bottlenecks in Healthcare Facilities and Coping Strategies Among Healthcare Workers in Niger. Environ Health Insights. 2024;18:11786302241271554. doi: 10.1177/11786302241271554 39148586 PMC11325333

[32] U.S. Mission Zambia. United States to Cut $50 Million in Medications and Medical Supplies Support [Internet]. 2025 [cited 2025 Aug 4]. Available from: https://zm.usembassy.gov/united-states-to-cut-50-million-in-medications-and-medical-supplies-support/

[33] Mulungu C, Ezekiel K, Kapungwe M. Facility preparedness for recurring cholera epidemics: A case of Matero and Kanyama sub-districts, in Lusaka. IJRISS. 2024;VIII(VII):1233–46.

[34] Gulumbe BH, Chishimba K, Shehu A, Chibwe M. Zambia’s battle against cholera outbreaks and the path to public health resilience: a narrative review. J Water Health. 2024;22(12):2257–75. 39733354 10.2166/wh.2024.094

[35] McInnes C, Lee K. Health, security and foreign policy. Rev Int Stud. 2006;32(1):5–23.

[36] Shears P, O’Dempsey TJD. Ebola virus disease in Africa: epidemiology and nosocomial transmission. J Hosp Infect. 2015;90(1):1–9. doi: 10.1016/j.jhin.2015.01.002 25655197

[37] Faye O, Boëlle P-Y, Heleze E, Faye O, Loucoubar C, Magassouba N, et al. Chains of transmission and control of Ebola virus disease in Conakry, Guinea, in 2014: an observational study. Lancet Infect Dis. 2015;15(3):320–6. doi: 10.1016/S1473-3099(14)71075-8 25619149 PMC4373532

[38] Frieden TR, Lee CT. Identifying and Interrupting Superspreading Events-Implications for Control of Severe Acute Respiratory Syndrome Coronavirus 2. Emerg Infect Dis. 2020;26(6):1059–66. doi: 10.3201/eid2606.200495 32187007 PMC7258476

[39] Auwal AR, Ishak AS, Saidu Musa S, Musa A, Saadu A, Riaz A. The global implications of U.S. withdrawal from WHO and the USAID shutdown: challenges and strategic policy considerations. Front Public Health. 2025;13. 10.3389/fpubh.2025.1589010 PMC12171363 40529700

[40] Kieny MP, Bekedam H, Dovlo D, Fitzgerald J, Habicht J, Harrison G. Strengthening health systems for universal health coverage and sustainable development. Bull World Health Organ. 2017;95(7):537–9. 28670019 10.2471/BLT.16.187476 PMC5487973

[41] US Government. U.S. Government Global Water Strategy 2022-2027. Washington, DC: United States Agency for International Development (USAID); 2022.

[42] United Nations Department of Economic and Social Affairs. The Sustainable Development Goals Report 2023: Special Edition. United Nations; 2023. (The Sustainable Development Goals Report).

[43] Kannampilly A, Sullivan A, Kannampilly A, Sullivan A. Exclusive: Trump’s funding cut stalls water projects, increasing risks for millions. Reuters [Internet]. 2025 [cited 2026 Jan 26]; Available from: https://www.reuters.com/world/us/trumps-funding-cut-stalls-water-projects-increasing-risks-millions-2025-07-20/

[44] Zambia Ministry of Health. 2022-2026 National Health Strategic Plan [Internet]. 2022. Available from: https://faolex.fao.org/docs/pdf/zam215377.pdf

[45] Chilufya C, Kamanga M. Crunch time: the transformational universal health coverage agenda for Zambia. Health Syst Reform. 2018;4(4):272–6. 30207824 10.1080/23288604.2018.1503031

[46] World Health Organization. Health financing progress matrix assessment, Zambia 2024: summary of findings and recommendations [Internet]. Geneva; 2024. Available from: https://iris.who.int/bitstream/handle/10665/378598/9789240098596-eng.pdf?sequence=1

[47] Osei Afriyie D, Masiye F, Tediosi F, Fink G. Purchasing for high-quality care using National Health Insurance: evidence from Zambia. Health Policy Plan. 2023;38(6):681–8. doi: 10.1093/heapol/czad022 37022137 PMC10274566

[48] Osei Afriyie D, Masiye F, Tediosi F, Fink G. Confidence in the health system and health insurance enrollment among the informal sector population in Lusaka, Zambia. Soc Sci Med. 2023;321:115750. 36801748 10.1016/j.socscimed.2023.115750

[49] Kombo S, Chulu D, Mudenda G. Factors Affecting Insurance Uptake in Zambia: A Case Study of Lusaka City.

[50] Hanson K, Brikci N, Erlangga D, Alebachew A, De Allegri M, Balabanova D, et al. The Lancet Global Health Commission on financing primary health care: putting people at the centre. Lancet Glob Health. 2022;10(5):e715–72. doi: 10.1016/S2214-109X(22)00005-5 35390342 PMC9005653

[51] Gichaga A, Masis L, Chandra A, Palazuelos D, Wakaba N. Mind the global community health funding gap. Glob Health Sci Pract. 2021;9(Supplement 1):S9–17. 10.9745/GHSP-D-20-00517 PMC7971370 33727316

[52] Rocks S, Berntson D, Gil-Salmerón A, Kadu M, Ehrenberg N, Stein V, et al. Cost and effects of integrated care: a systematic literature review and meta-analysis. Eur J Health Econ. 2020;21(8):1211–21. doi: 10.1007/s10198-020-01217-5 32632820 PMC7561551

[53] Wang X, Yang E, Zheng C, Yuan S. Effects of vertical integration on the healthcare system in China: a systematic review and meta-analysis. Health Policy Plan. 2024;39(1):66–79. 37768012 10.1093/heapol/czad085 PMC10775222

[54] Sakala JJ, Chimatiro CS, Salima R, Kapachika A, Kalepa J, Stones W. The integration of vertical and horizontal programmes for health systems strengthening in Malawi: a case study. Mal Med J. 2022;34(3):206–12. 10.4314/mmj.v34i3.11 PMC9641613 36406101

[55] Peters DH, Paina L, Schleimann F. Sector-wide approaches (SWAps) in health: what have we learned?. Health Policy Plan. 2013;28(8):884–90. 23236010 10.1093/heapol/czs128

[56] Richardson AK. Investing in public health: barriers and possible solutions. J Public Health (Oxf). 2012;34(3):322–7. doi: 10.1093/pubmed/fds039 22696599

[57] Mutale W, Bosomprah S, Shankalala P, Mweemba O, Chilengi R, Kapambwe S, et al. Assessing capacity and readiness to manage NCDs in primary care setting: Gaps and opportunities based on adapted WHO PEN tool in Zambia. PLoS One. 2018;13(8):e0200994. doi: 10.1371/journal.pone.0200994 30138318 PMC6107121

[58] Kemei J, Etowa J. ‘It Is Beyond Our Reach’: Policies and Infrastructure Influencing Postpartum Care in Rural Kenya. Research Square [Internet]. 2021 [cited 2022 Oct 18]. Available from: https://www.researchsquare.com/article/rs-781739/v1

[59] Fitzpatrick T, Tantum LK, Tseka JM, Mofolo I, Kafanikhale H, Hoffman I, et al. The effects of environmental health services on patient well-being and quality of care: A qualitative study in Malawi’s public healthcare facilities. SSM Health Syst. 2025;4:100064. doi: 10.1016/j.ssmhs.2025.100064 40980020 PMC12445730

[60] Thaddeus S, Maine D. Too far to walk: maternal mortality in context. Soc Sci Med. 1994;38(8):1091–110. doi: 10.1016/0277-9536(94)90226-7 8042057

[61] Bouzid M, Cumming O, Hunter PR. What is the impact of water sanitation and hygiene in healthcare facilities on care seeking behaviour and patient satisfaction? A systematic review of the evidence from low-income and middle-income countries. BMJ Glob Health. 2018;3(3):e000648. doi: 10.1136/bmjgh-2017-000648 29765776 PMC5950627

[62] Whetten K, Leserman J, Whetten R, Ostermann J, Thielman N, Swartz M, et al. Exploring lack of trust in care providers and the government as a barrier to health service use. Am J Public Health. 2006;96(4):716–21. doi: 10.2105/AJPH.2005.063255 16507725 PMC1470533

[63] Tsai LL, Morse BS, Blair RA. Building credibility and cooperation in low-trust settings: persuasion and source accountability in Liberia during the 2014–2015 Ebola crisis. Comp Polit Stud. 2020;53(10–11):1582–618.

[64] Clark RA, McQuaid CF, Richards AS, Bakker R, Sumner T, Prŷs-Jones TO, et al. The potential impact of reductions in international donor funding on tuberculosis in low-income and middle-income countries: a modelling study. The Lancet Global Health [Internet]. 2025 [cited 2025 Jul 28]; Available from: https://linkinghub.elsevier.com/retrieve/pii/S2214109X25002323 10.1016/S2214-109X(25)00232-3 40659023

[65] Kyobutungi C, Okereke E, Abimbola S. After USAID: what now for aid and Africa?. BMJ. 2025;r479. 10.1136/bmj.r479 40068845

